# The NAMPT inhibitor FK866 reverts the damage in spinal cord injury

**DOI:** 10.1186/1742-2094-9-66

**Published:** 2012-04-10

**Authors:** Emanuela Esposito, Daniela Impellizzeri, Emanuela Mazzon, Gohar Fakhfouri, Reza Rahimian, Cristina Travelli, Gian Cesare Tron, Armando A Genazzani, Salvatore Cuzzocrea

**Affiliations:** 1 Department of Clinical and Experimental Medicine and Pharmacology, School of Medicine, University of Messina, Torre Biologica - Policlinico Universitario Via C. Valeria Gazzi, 98100 Messina, Italy; 2 Fondazione Umberto Veronesi, P.zza Velasca 5, Milano 20122, Italy; 3 IRCCS Centro Neurolesi "Bonino-Pulejo", Messina 98124, Italy; 4 Department of Pharmacology, School of Medicine, Shahid Beheshti University of Medical Sciences, Tehran, Iran; 5 Department of Pharmacology, School of Medicine, Tehran University of Medical Sciences, Tehran, Iran; 6 DiSCAFF, Università del Piemonte Orientale, Novara 28100, Italy

**Keywords:** NAMPT inhibitor, Spinal cord injury, Inflammation, Cytokines, Apoptosis, Neurotrophic factors

## Abstract

**Background:**

Emerging data implicate nicotinamide phosphoribosyl transferase (NAMPT) in the pathogenesis of cancer and inflammation. NAMPT inhibitors have proven beneficial in inflammatory animal models of arthritis and endotoxic shock as well as in autoimmune encephalitis. Given the role of inflammatory responses in spinal cord injury (SCI), the effect of NAMPT inhibitors was examined in this setting.

**Methods:**

We investigated the effects of the NAMPT inhibitor FK866 in an experimental compression model of SCI.

**Results:**

Twenty-four hr following induction of SCI, a significant functional deficit accompanied widespread edema, demyelination, neuron loss and a substantial increase in TNF-α, IL-1β, PAR, NAMPT, Bax, MPO activity, NF-κB activation, astrogliosis and microglial activation was observed. Meanwhile, the expression of neurotrophins BDNF, GDNF, NT3 and anti-apoptotic Bcl-2 decreased significantly. Treatment with FK866 (10 mg/kg), the best known and characterized NAMPT inhibitor, at 1 h and 6 h after SCI rescued motor function, preserved perilesional gray and white matter, restored anti-apoptotic and neurotrophic factors, prevented the activation of neutrophils, microglia and astrocytes and inhibited the elevation of NAMPT, PAR, TNF-α, IL-1β, Bax expression and NF-κB activity.

We show for the first time that FK866, a specific inhibitor of NAMPT, administered after SCI, is capable of reducing the secondary inflammatory injury and partly reduce permanent damage. We also show that NAMPT protein levels are increased upon SCI in the perilesional area which can be corrected by administration of FK866.

**Conclusions:**

Our findings suggest that the inflammatory component associated to SCI is the primary target of these inhibitors.

## Background

Nicotinamide phosphoribosyl transferase (NAMPT) is an enzyme that catalyzes the synthesis of nicotinamide mononucleotide (NMN) from nicotinamide (NM) and 5'-phosphoribosyl-1'-pyrophosphate (PRPP), thus playing an important role in the cyclic biosynthetic pathway of nicotinamide adenine dinucleotide (NAD). As such, this enzyme is central to cellular bioenergetics, and may control indirectly a number of signalling pathways that depend on NAD levels, such as Poly (ADP-ribose) polymerase (PARP) and sirtuin activation [1]. A secreted form has been described, which is also known as visfatin or Pre-B cell colony-enhancing factor (PBEF) [2]. Indeed, it has also been postulated that NAMPT behaves as an adipokine secreted from visceral fat tissues [3] or may have an important role in immune system. For example, NAMPT has been originally identified as an extracellular proinflammatory cytokine, able to induce cellular expression of inflammatory cytokines such as tumor necrosis factor (TNF)-α, interleukin (IL)-1β and IL-6 and to promote pre-B cell colony formation [4,5]. Lymphocytes, dendritic cells, monocytes and macrophages express NAMPT when presented inflammatory stimuli [6-8]. This suggests that NAMPT, either via the "NAD salvage pathway" or by other unknown mechanisms relating to its secreted form, may modulate innate or acquired immune functions.

Emerging data implicate PBEF/NAMPT/Visfatin in the pathogenesis of a number of different human diseases, in particular in the field of cancer and inflammation [3]. The link between NAMPT and inflammation is rapidly strengthening. Indeed, visfatin levels have been found elevated in the systemic circulation of patients suffering from diseases with inflammatory components, such as type 2 diabetes [9], acute pancreatitis [10], osteoarthritis [11], sepsis [12], atherosclerosis [13] and psoriasis [14].

The search for novel antitumoral drugs has led to the identification of two inhibitors of NAMPT, FK866 (also known as APO866) and CHS828, which have now entered Phase II clinical trials. Given the role of NAMPT in inflammatory processes, an important question is whether these agents may protect from inflammatory damage. While *in vitro *evidences would support the use of these drugs in inflammatory processes [12,15], *in vivo *evidence is limited. Indeed, it has been shown that FK866 is able to reduce disease burden in inflammatory animal models of arthritis and endotoxic shock [15,16] as well as in experimental autoimmune encephalitis [17]. These studies have highlighted that NAMPT inhibitors exert their effects in a pleiotropic manner, by reducing cytokine release, as well as offsetting PARP and sirtuin activation.

Spinal cord injury (SCI) is a highly debilitating pathology [18]. Although innovative medical care has improved patient outcome, advances in pharmacotherapy for minimizing neuronal injury and promoting regeneration are limited. The complex pathophysiology of SCI may explain the difficulty in finding a suitable therapy. The primary traumatic mechanical injury to the SC causes the death of a number of neurons that cannot be regenerated: neurons continue to die for hours following traumatic SCI [19]. The events that characterize this successive phase to mechanical injury are called "secondary damage" characterized by cellular, molecular, and biochemical cascades. The presence of a local inflammatory response maintains and amplifies the secondary damage [20]. When SCI occurs, microglia in parenchyma is activated and macrophages in circulation get across blood-brain barrier (BBB) to act as intrinsic spinal phagocytes. These cells release different pro-inflammatory mediators such as proinflammatory cytokines [21] and reactive oxygen species (ROS) and nitrogen species [22]. Nitric oxide (NO) produced by inducible nitric oxide synthase (iNOS) modulate the secondary inflammatory response following traumatic SCI [23]. ROS and peroxynitrite also cause DNA damage, which results in the activation of the nuclear enzyme PARP, depletion of NAD^+ ^and ATP and ultimately cell death [24]. Therefore, recently it has been demonstrated that SCI induced PARP activation [25].

In the light of these evidences and given the high therapeutic unmet need of SCI, we investigated the possible contribution of NAMPT in this condition. We now show that pharmacological inhibition of NAMPT given after the injury leads to a significant reduction of inflammatory cytokines, a histological improvement of the perilesional area and a significant recovery of locomotor activity.

## Methods

### Animals

Male Adult CD1 mice (25-30 g, Harlan, Milan, Italy) were housed in a controlled environment. Animal care was in compliance with Italian regulations on protection of animals used for experimental and other scientific purpose (DM 116192) as well as with the EEC regulations (DL 116/92, application of the European Communities Council Directive 86/609/EEC). All efforts were made to minimize animal suffering and the number of animals used.

### SCI

We used the clip compression model described by Rivlin and Tator [26]. Mice were anesthetized using chloral hydrate (400 mg/kg body weight). A longitudinal incision was made on the midline of the back, exposing the paravertebral muscles. These muscles were dissected away exposing T5-T8 vertebrae. The spinal cord was exposed via a four-level T5-T8 laminectomy and SCI was produced by extradural compression at T6-T7 level using an aneurysm clip with a closing force of 24 g. In all injured groups, the spinal cord was compressed for 1 min. Sham animals were only subjected to laminectomy. Following surgery, 1.0 cc of saline was administered subcutaneously in order to replace the blood volume lost during the surgery. During recovery from anesthesia, the mice were placed on a warm heating pad and covered with a warm towel. The mice were individually housed in a temperature-controlled room at 27°C for a survival period of 20 days. Food and water were provided to the mice ad libitum. During this time period, the animals' bladders were manually voided twice a day until the mice were able to regain normal bladder function.

### Experimental groups and treatments

Mice were randomly allocated into the following groups: (i) sham + vehicle group. Mice were subjected to laminectomy but the aneurysm clip was not applied, and treated intraperitoneally with vehicle (N = 30). (ii) Sham + FK866 group. Identical to sham + vehicle group except for intraperitoneal administration of FK866 (10 mg/kg) 1 h and 6 h after laminectomy (N = 30). (iii) SCI + vehicle group. Mice were subjected to SCI and were administered vehicle at 1 h and 6 h after SCI (N = 30). (iv) SCI + FK866 group. Mice were subjected to SCI and administered FK866 (10 mg/kg, intraperitoneally) at 1 h and 6 h after SCI (N = 30).

Timing of administration of FK866 was similar to that proposed for methylprednisolone at high concentrations (the current therapy available for traumatic SCI). FK866 were synthesized as reported [27]. The dose of FK866 used here was based on previous *in vivo *studies [15-17]. Alongside, all experiments were also performed with an analogue of FK866, named GPP78, and data were super-imposable to those obtained with FK866 (data not shown).

In a separate set of experiments to investigate the motor score, additional animals (10 animals/each group) were observed until 20 days after SCI. FK866 (10 mg/kg) was administered 1 h and 6 h after SCI and daily until day 19.

### Tissue processing

At the end of the experimental period (24 hrs), animals were deeply anesthetized with sodium pentobarbital and then perfused transcardially with cold phosphate-buffered saline (PBS, 0.1 M) followed by 4% paraformaldehyde in 0.1 M PBS, pH 7.4. SC tissues were removed under magnified vision. Tissue segments containing the lesion (1 cm on each side of the lesion) were paraffin embedded, cut into longitudinal sections for posterior area of SC, and processed for different immunohistochemical procedures.

### Different tissues were also collected for Golgi-Cox staining, which highlights neurons and processes

For immunoblotting analysis, mice (N = 10) were anaesthetized and exsanguinated via cardiac puncture. SCs were dissected, and cleaned from meninges and nerve roots. One centimeter of the cord centered at the injury site was homogenized for preparing cytosolic and nuclear extracts.

### Myeloperoxidase activity

Myeloperoxidase (MPO) activity, an indicator of polymorphonuclear leukocyte (PMN) accumulation, was determined in SC tissues as previously described [28]. MPO activity was expressed in units/mg protein.

### Measurement of TNF-α and IL-1β

SC tissues, collected at 24 hrs after SCI, were homogenized in PBS containing 2 mmol/L of phenyl-methyl sulfonyl fluoride and tissue TNF-α and IL-1β levels were evaluated by using a colorimetric commercial kit DuoSet ELISA Development System (R&D Systems, Milan, Italy).

### Immunohistochemical localization of Bax, Bcl-2, GFAP, CD11-β, BDNF, GDNF, NT-3, PAR, and NAMPT

Sections (8 μm) were prepared from paraffin embedded tissues. After deparaffinization, endogenous peroxidase was quenched with 0.3% hydrogen peroxide in 60% methanol for 30 minutes. The sections were permeabilized with 0.1% Triton X-100 in PBS for 20 minutes. Sections were incubated overnight with anti-Bax (1:500; Santa Cruz Biotechnology, Santa Cruz, CA, USA), or anti-Bcl-2 (1:500, Santa Cruz Biotechnology, Santa Cruz, CA, USA), anti-GFAP (1:500, Santa Cruz Biotechnology, Santa Cruz, CA, USA), anti-CD11-β (1:500; AbD Serotec, Raleigh, NC, USA), anti-BDNF (1:500, Santa Cruz Biotechnology, Santa Cruz, CA, USA), anti-GDNF (1:500, Santa Cruz Biotechnology, Santa Cruz, CA, USA), anti-NT3 (1:500, Santa Cruz Biotechnology, Santa Cruz, CA, USA), anti-PAR (10 μg/ml; Merck Millipore, Billerica, MA, USA), and NAMPT (10 μg/ml; Novus Biologicals, Littleton, CO, USA). Sections were incubated with secondary antibody. Specific labeling was detected with a biotin-conjugated goat anti-rabbit IgG and avidin-biotin peroxidase complex (Vector Lab. Inc., Burlingame, CA, USA). Immunocytochemistry photographs (n = 5 photos from each samples collected from all mice in each experimental group) were assessed by densitometry as previously described [29,30] by using Optilab (Graftek, France) software on a Macintosh personal computer (CPU G3-266; Apple, Inc., Cupertino, CA, USA).

### Golgi-Cox staining

The tissues were fixed in 10% buffered formalin, and placed on a layer of glass wool in a large volume of Golgi-Cox solution and incubated at 37°C. After two months of incubation, the tissue samples were washed, dehydrated, cleared, wax impregnated and ultimately embedded in molten paraffin wax. 50 μ-thick sections were cut with the help of rotary microtome. The sections were collected in warm water and then mounted on albumenised slides. The slides were kept in oven at 50°C for half an hour. The paraffin wax was removed by immersing the slides in xylene. The slides were passed through descending grades of alcohol and ultimately dipped in distilled water. Blackening was done by placing the sections in 5% ammonia solution for one hour and subsequently washed thoroughly in distilled water. The tissue sections were dehydrated, cleared and mounted.

#### Light microscopy

Section 5 μm-thick sections were deparaffinized with xylene, stained with Haematoxylin/Eosin (H&E), with Luxol Fast Blue with cresyl violet (Klüver and Barrera stain) counterstaining (used to assess demyelination) and studied using light microscopy (Dialux 22 Leitz, Milan Italy). Damaged neurons were counted and the histopathologic changes of the gray matter were scored on a 6-point scale [31]: 0, no lesion observed, 1, gray matter contained 1 to 5 eosinophilic neurons; 2, gray matter contained 5 to 10 eosinophilic neurons; 3, gray matter contained more than 10 eosinophilic neurons; 4, small infarction (less than one third of the gray matter area); 5, moderate infarction; (one third to one half of the gray matter area); 6, large infarction (more than half of the gray matter area). The scores from all the sections from each spinal cord were averaged to give a final score for an individual mice.

### Terminal Deoxynucleotidyltransferase-Mediated UTP End Labeling (TUNEL) Assay

TUNEL assay was conducted by using a TUNEL detection kit according to the manufacturer's instruction (Apotag, HRP kit DBA, Milan, Italy), as previously reported [32]. The signals were visualized with diaminobenzidine.

### Preparation of spinal cord extracts and Western blot analysis for phospho-NF-κB p65 (serine 536), NF-κB p65, Bax and Bcl-2

Cytosolic and nuclear extracts were prepared as previously described [33] with slight modifications. The filters were blocked with 5% non-fat dried milk (AppliChem GmbH, Germany) in PBS (PM) for 40 minutes at room temperature (RT) and subsequently probed with specific Abs phospho-NFκB p65 (serine 536) (Cell Signaling, 1:1,000), or anti-Bax (1:500; Santa Cruz Biotechnology, Santa Cruz, CA, U.S.A.), or anti-Bcl-2 (1:500; Santa Cruz Biotechnology, Santa Cruz, CA, U.S.A.), or anti-NFκB p65 (1:1,000; Santa Cruz Biotechnology, Santa Cruz, CA, U.S.A.) in PM with 0.1% Tween-20 (Sigma-Aldrich, Milan, Italy) (PMT) at 4°C, overnight. Membranes were incubated with peroxidase-conjugated bovine anti-mouse IgG secondary antibody or peroxidase-conjugated goat anti-rabbit IgG (1:2,000, Jackson ImmunoResearch, West Grove, PA, USA) for 1 h at RT. To ascertain that blots were loaded with equal amounts of protein lysates, they were also incubated in the presence of the Abs against β-actin and lamin A/C (1:10,000 Sigma-Aldrich, Milan, Italy). The relative expression of the protein bands of phospho NFκB (75 kDa), NFκB p65 (65 kDa), Bax (approximately 23 kDa), Bcl-2 (approximately 29 kDa) was quantified by densitometric scanning of the X-ray films with Imaging Densitometer (GS-700, Bio-Rad Laboratories, Milan, Italy) and a computer program (Molecular Analyst, IBM, Milan, Italy).

### Grading of motor disturbance

The motor function of mice subjected to compression trauma was assessed once a day for 20 days after injury. Recovery from motor disturbance was graded using the Basso Mouse Scale (BMS) open-field score [34], since the BMS has been shown to be a valid locomotor rating scale for mice. The evaluations were made by two blind observers for all analyzed groups. Briefly, the BMS is a nine-point scale that provides a gross indication of locomotor ability and determines the phases of locomotor recovery and features of locomotion. The BMS scale ranges from 0 (indicating complete paralysis) to 9 (indicating normal hindlimb function), rating locomotion on aspects of hindlimb function such as weight support, stepping ability, coordination, and toe clearance. The BMS score was determined for ten mice in each group.

## Materials

All compounds, except for FK866 and GPP78, were obtained from Sigma-Aldrich (Milan, Italy). FK866 was synthesized in house as described previously and was over 99% pure [35]. All chemicals were of the highest commercial grade available. All stock solutions were prepared in non-pyrogenic saline (0.9% NaCl; Baxter, Italy, UK).

### Statistical evaluation

All values in the figures and text are expressed as mean ± standard error of the mean (SEM) of N observations. For the *in vivo *studies N represents the number of animals studied. In the experiments involving histology or immunohistochemistry, the figures shown are representative of at least 3 experiments performed on different experimental days. The results were analyzed by one-way ANOVA followed by a Bonferroni *post-hoc *test for multiple comparisons. A *p*-value of less than 0.05 was considered significant. BMS scale data were analyzed by the Mann-Whitney test and considered significant when p-value was < 0.05

## Results and discussion

### FK866 and GPP78 treatments reduce the severity of spinal cord trauma

**Figure 1:**
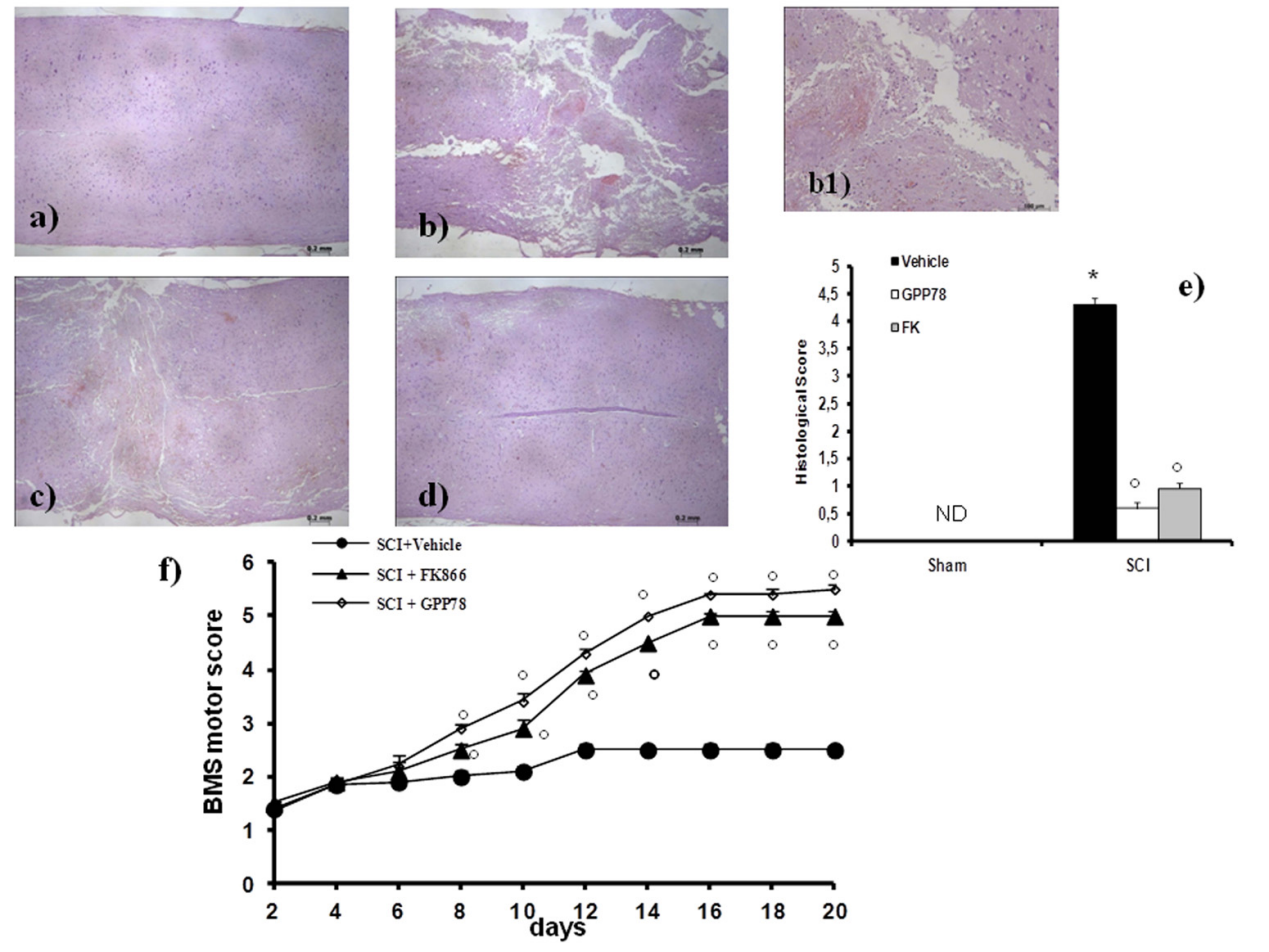
**Effects of FK866 treatment on histological alterations of the spinal cord tissue 24 h after injury and motor function**. Significant damage to the spinal cord in mice subjected to SCI, at the perilesional area, was apparent, as evidenced by the presence of edema as well as alteration of the white matter 24 h after injury (**b, b1**). Notably, an important protection from SCI-associated damage was observed in the tissue samples collected from FK866 (**c**) treated mice. GPP78 (**d**) displayed the same effects of FK866 in histological score (**e**) and similar improvements in the motor activity (**f**). The histological score (**e**) was made by an independent observer. The degree of motor disturbance was assessed every day until 20 days after SCI by BMS criteria (**f**). Treatments with FK866 enhanced the recovery after SCI. This figure is representative of at least 3 experiments performed on different experimental days. Data are means ± s.e. means of 10 mice for each group. *p < 0.01 vs. Sham. °*p *< 0.01 vs SCI + vehicle.

**Figure 2:**
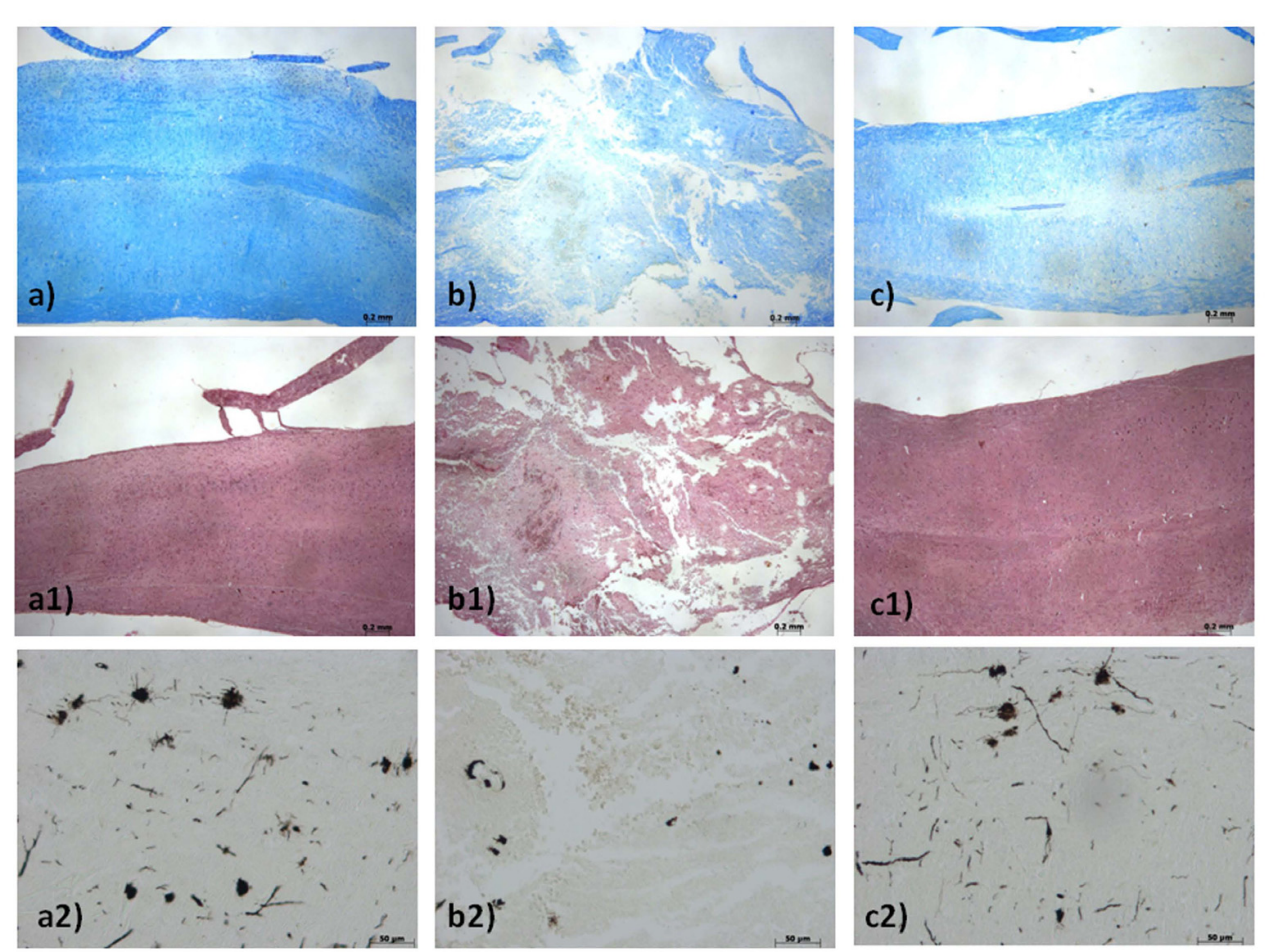
**Effects of FK866 treatment on myelin structure alteration**. Myelin structure was observed by Luxol fast blue staining. At 24 h after the injury in SCI operated mice (**b**) a loss of myelin was observed. In contrast in FK866 (**c**) treated mice myelin degradation was attenuated. No significant alterations was observed in sections obtained by sham groups (**a**). Moreover Weigert's and Oil Red staining is highly specific for degenerating myelin and for myelin that has been ingested by macrophages. At 24 h after injury, in SCI mice group was observed a greater density of Oil Red staining as a marker for myelin destruction (**b**1) when compared with control mice (**a1**). In contrast in FK866 (**c1**) treated mice myelin degradation was attenuated. In order in the spinal cord tissue collected from SCI mice, was observed the alteration of morphology of neurons when compared with sham-operated mice (**b2**). A protection against alteration of neuron's morphology was observed in mice group treated with FK866 (**c2**). This figure is representative of at least 3 experiments performed on different experimental days.

The severity of the trauma at the level of the perilesional area, assessed by the presence of edema as well as alteration of the white matter and infiltration of leukocytes, was evaluated 24 h after injury by hematoxylin/eosin (H&E) staining. Significant damage was observed in the spinal cord tissue collected from SCI (Figure 1b) when compared with sham-operated mice (Figure 1a). Protection against the SCI was observed in FK866-treated mice (Figure 1c). Moreover, to evaluate the severity of the trauma we also investigated the alteration in myelination. In sham-treated mice, as expected, myelin appeared normal (Figure 2a). On the contrary, a significant alteration was detected in the spinal cord tissues collected at 24 hours after SCI (Figure 2b). Treatment with FK866 (Figure 2c) significantly reduced the demyelination associated with SCI. The histological score was evaluated by an independent observer and confirmed these findings (Figure 1e). In order to evaluate if histological damage to the spinal cord was associated with a loss of motor function, the BMS hind limb locomotor rating scale score was evaluated. Mice subject to SCI had significant deficits in movement (Figure 1f). Treatment of animals with FK866 (Figure 1f) significantly ameliorated the functional deficits induced by SCI. To confirm these effects, we took advantage of a recently synthesized analogue of FK866, named GPP78 [35]. Indeed, GPP78 displayed the same effects in histological score and similar improvements in the motor activity of SCI animals (Figures 1d, e, f).

### FK866 modulates cytokines expression and neutrophil infiltration after SCI

**Figure 3:**
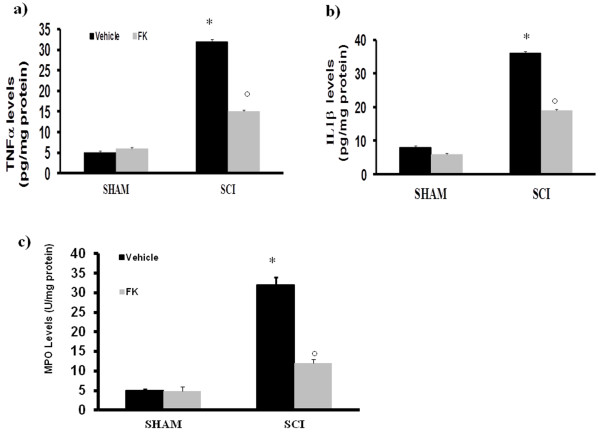
**Effects of FK866 on TNF-α and IL-1β release and on MPO activity**. A substantial increase in TNF-α (**a**) and IL-1β (**b**) production was found in spinal cord tissue collected from SCI mice at 24 h. Spinal cord levels of TNF-α and IL-1β were significantly attenuated by the FK866. Moreover, MPO activity in spinal cord of untreated SCI-operated mice was significantly increased at 24 h after the damage in comparison to sham mice (**c**). Treatment with FK866 significantly reduced the SCI-induced increase in MPO activity. Data are means ± S.E.M of 10 mice for each group. *p < 0.01 vs sham, °*p *< 0.01 versus SCI + vehicle.

As our aim was to understand the mechanisms by which NAMPT inhibitors exert this effect, we decided to concentrate solely on the most established of the two pharmacological tools, FK866. To test whether FK866 modulates the inflammatory process through the regulation of secretion of pro-inflammatory cytokines, we analyzed spinal cord tissue levels of TNF-α and IL-1β. A substantial increase in TNF-α and IL-1β production was found in spinal cord samples collected from SCI mice 24 h after SCI (Figure 3a and 3b respectively). Spinal cord levels of TNF-α and IL-1β were significantly attenuated by the administration of FK866 (Figure 3a). The histological pattern of SCI seemed to be correlated with the influx of leukocytes into the spinal cord. Therefore, we investigated the effect of the treatments on the infiltration of neutrophils by measuring myeloperoxidase (MPO), a lysosomal protein stored in azurophilic granules of the neutrophil (Figure 3c). MPO activity was significantly elevated in the spinal cord at 24 h after injury in mice subjected to SCI when compared with Sham-operated mice (Figure 3c). In FK866-treated mice, the activity of this peroxidase enzyme was significantly attenuated (Figure 3c) in comparison to that observed in SCI.

### Effect of FK866 on phosphorylation of p65 on Ser536 and nuclear NF-κB p65

**Figure 4:**
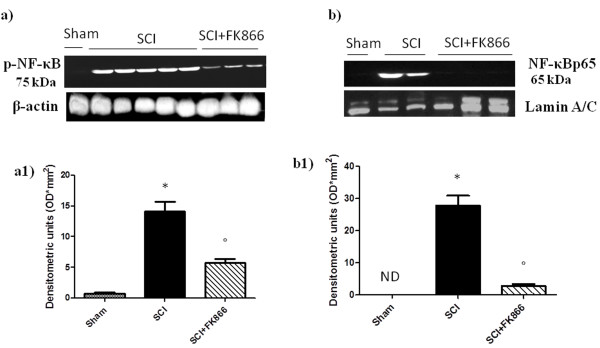
**Effects of FK866 treatment on NF-κB p65 activation**. By Western blot analysis, we evaluated the phosphorylation of Ser536 on the NF-κB subunit p65 and nuclear NF-κB p65 to investigate the cellular mechanisms by which treatments may attenuate the development of SCI. SCI caused a significant increase in the phosphorylation of NF-κB p65 on Ser536 at 24 h after the injury (**a, a1**); instead the treatment with FK866 prevented significantly the activation of NF-κB (**a, a1**). Moreover, traslocation of NF-κB p65 subunit in the nuclear fractions of the spinal cord tissue were also significant increased at 24 h after SCI compared with the sham-operated mice (**b, b1**). The levels of NF-κB p65 protein were significantly reduced in the nuclear fractions of the spinal cord tissues from animals that had received FK866 treatment as shown in (**b, b1**). Immunoblotting in a, b are representative of one spinal cord of five analyzed. The results in a1 and b1 are expressed as mean ± S.E.M from five blots, *p < 0.01 vs sham, °*p *< 0.01 versus SCI + vehicle.

By Western blot analysis, we evaluated the phosphorylation of Ser536 on the NF-κB subunit p65 and nuclear NF-κB p65 to investigate the cellular mechanisms by which treatments may attenuate the development of SCI. SCI caused a significant increase in the phosphorylation of NF-κB p65 on Ser536 at 24 h after the injury (Figure 4a and a1); treatment with FK866 prevented the activation of NF-κB (Figure 4a and a1). In addition, the translocation of NF-κBp65 was also significantly increased at 24 h after SCI compared with the sham-operated mice (Figure 4b and b1). FK866 treatment significantly reduced the levels of nuclear NF-κB p65 protein as shown (Figure 4b and b1).

Instead, IκB-α expression did not seem to be affected by FK866 treatment at 24 h after the injury (data not shown).

### Effect of FK866 on NAMPT and PAR formation

**Figure 5:**
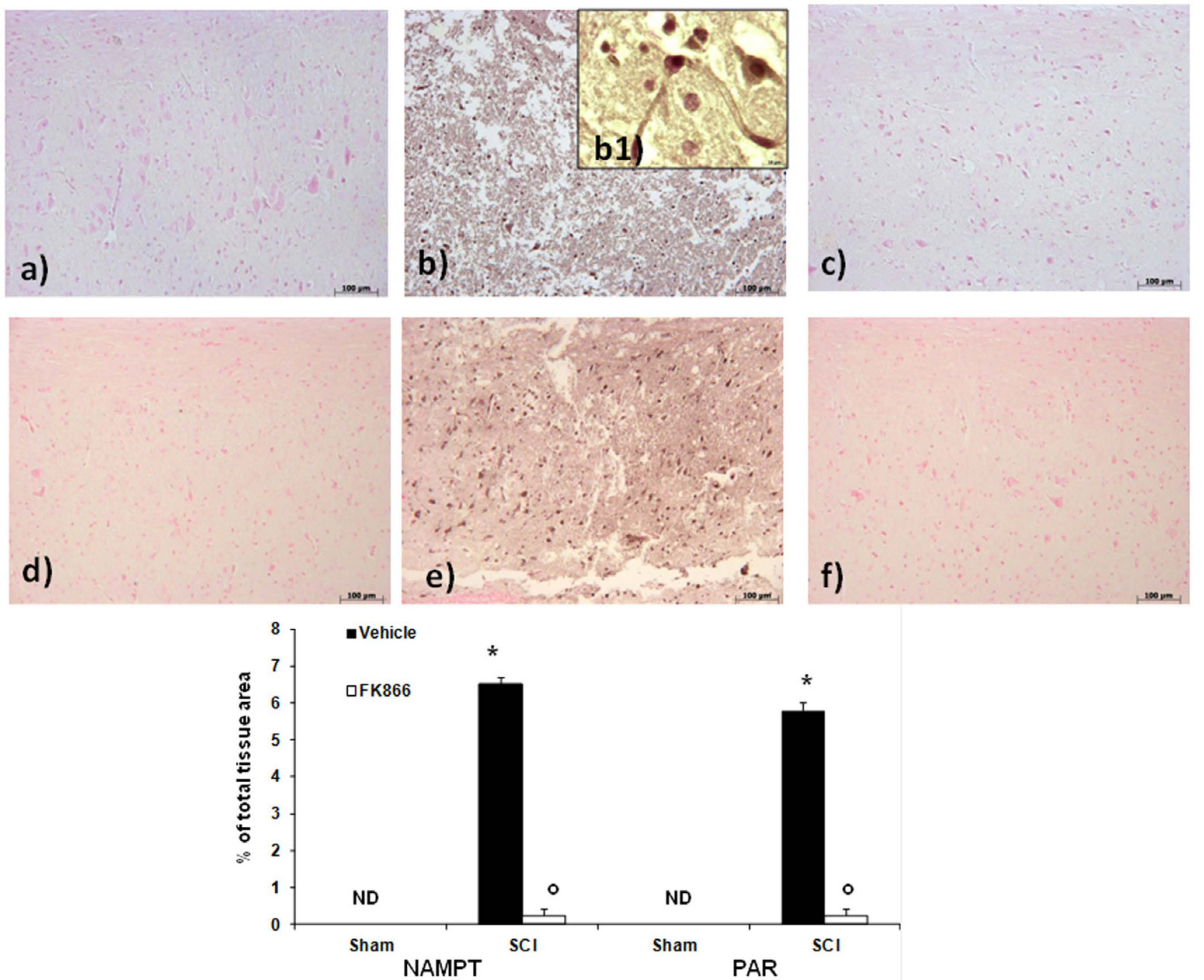
**Effects of FK866 on NAMPT and PAR formation**. NAMPT and PAR formation was evaluated by immunohistochemical analysis in the spinal cord section at 24 h after SCI. Spinal cord section obtained from SCI animals demonstrated positive staining for NAMPT in the white and gray matter (**b, see the particular b1**). FK866 treatment reduced the degree for positive staining for NAMPT in the spinal cord tissue (**c**). Moreover, immunoistrochemistry for PAR revealed the occurrence of positive staining for PAR in mice subject to SCI (**e**). FK866 treatment reduced the degree for positive staining for PAR in the spinal cord tissue (**f**). This figure is representative of at least 3 experiments performed on different experimental days.

Nicotinamide phosphoribosyl transferase activity has been shown to be essential for maintaining adequate intracellular NAD levels, influencing biological responses such as cell survival and inflammation. We then evaluated NAMPT presence with immunohistochemistry staining. We reveled a positive staining in the spinal cord tissues from mice subjected to SCI (Figure 5b, b1, see densitometric analysis).

FK866 decreased the degree of positive staining for NAMPT in the spinal cord from mice subjected to SCI (Figure 5c). Moreover, immunohistochemistry for PAR, as an indicator of *in vivo *PARP activation, revealed the occurrence of positive staining for PAR localized in nuclei of Schwann cells in the white and gray matter of the spinal cord tissues from mice subjected to SCI (Figure 5e). FK866 treatment reduced the degree of positive staining for PAR (Figure 5f) in the spinal cord.

### Effect of FK866 on apoptosis in spinal cord after injury

To test whether the tissue damage was associated with the induction of apoptosis, we evaluated at 24 h after injury, the possible changes in apoptotic proteins (Bax and Bcl-2) and the DNA fragmentation (TUNEL-like staining).

**Figure 6:**
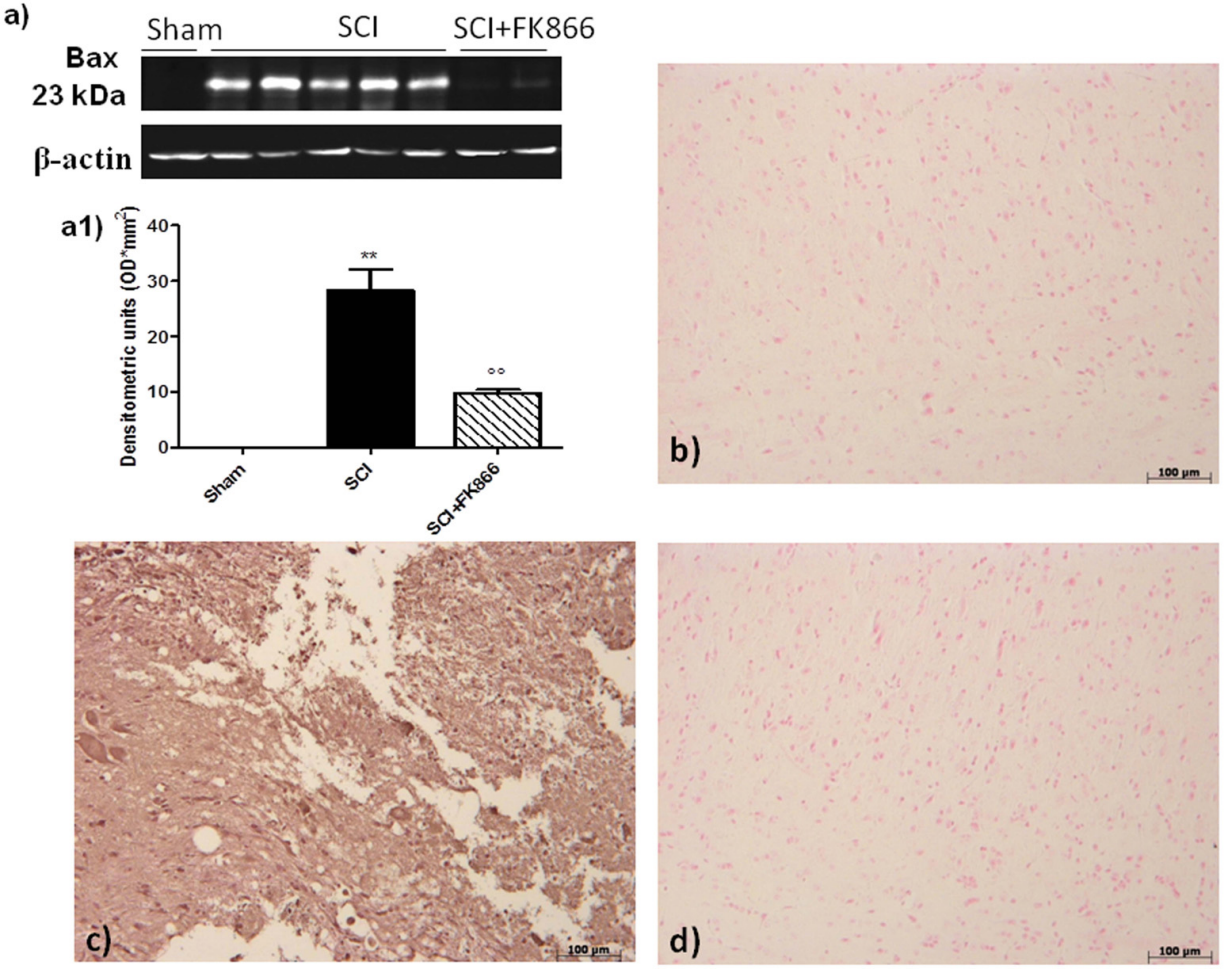
**Effects of FK866 on Bax expression**. By western blot and immunohistochemical analysis Bax levels were evaluated. Spinal cord obtained from SCI-operated mice exhibited appreciably increased Bax levels (**a, a1 and c**). FK866 (**a, a1 and d**) treatment prevented the SCI-induced Bax expression. The relative expression of the protein bands was standardized for densitometric analysis to β-actin levels, reported in panel a, a1 and are expressed as mean ± S.E.M from n = 5/6 spinal cord for each group. **p < 0.001 vs sham, °°*p *< 0.001 vs SCI + vehicle.

Spinal cord tissue was taken at 24 h after SCI in order to determine the immunohistological staining for Bax and Bcl-2. Spinal cord sections from sham-operated mice did not stain for Bax (Figure 6b, see densitometric analysis 7e) whereas spinal cord sections obtained from SCI mice (Figure 6c) exhibited a strong staining for Bax. Treatment with FK866 (Figure 6d) decreased the degree of positive staining for Bax in the spinal cord from mice subjected to SCI.

**Figure 7:**
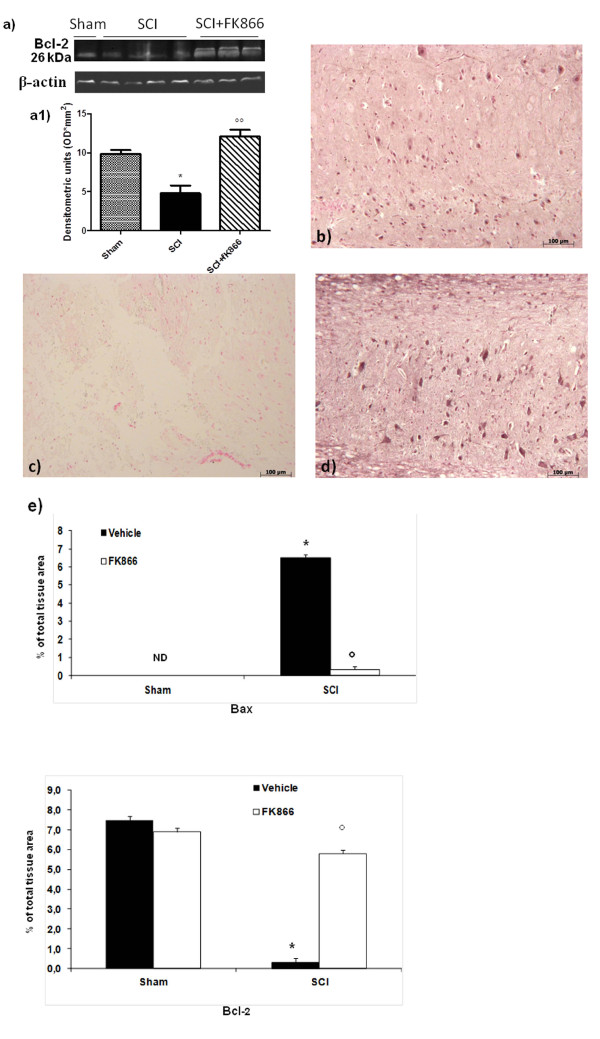
**Effects of FK866 on Bcl-2 expression**. Moreover, a basal level of Bcl-2 expression was detected in spinal cord from sham-operated mice (**a, a1 and b**). Twenty-four hours after SCI, Bcl-2 expression was significantly reduced in spinal cord from SCI mice (**a, a1 and c**). Treatment with FK866 (**a, a1 and d**) significantly reduced the SCI-induced inhibition of Bcl-2 expression. The relative expression of the protein bands was standardized for densitometric analysis to β-actin levels, reported in panel a, a1 and are expressed as mean ± S.E.M from n = 5/6 spinal cord for each group. *p < 0.01 vs sham, °°*p *< 0.001 vs SCI + vehicle.

In addition, spinal cord from sham-operated mice demonstrated positive staining for Bcl-2 (Figure 7b) while in SCI mice the staining for Bcl-2 was significantly reduced (Figure 7c). FK866 (Figure 7d and 7e) treatments restored the levels of this anti-apoptotic protein. Similar results were obtained by western blot analysis.

**Figure 8:**
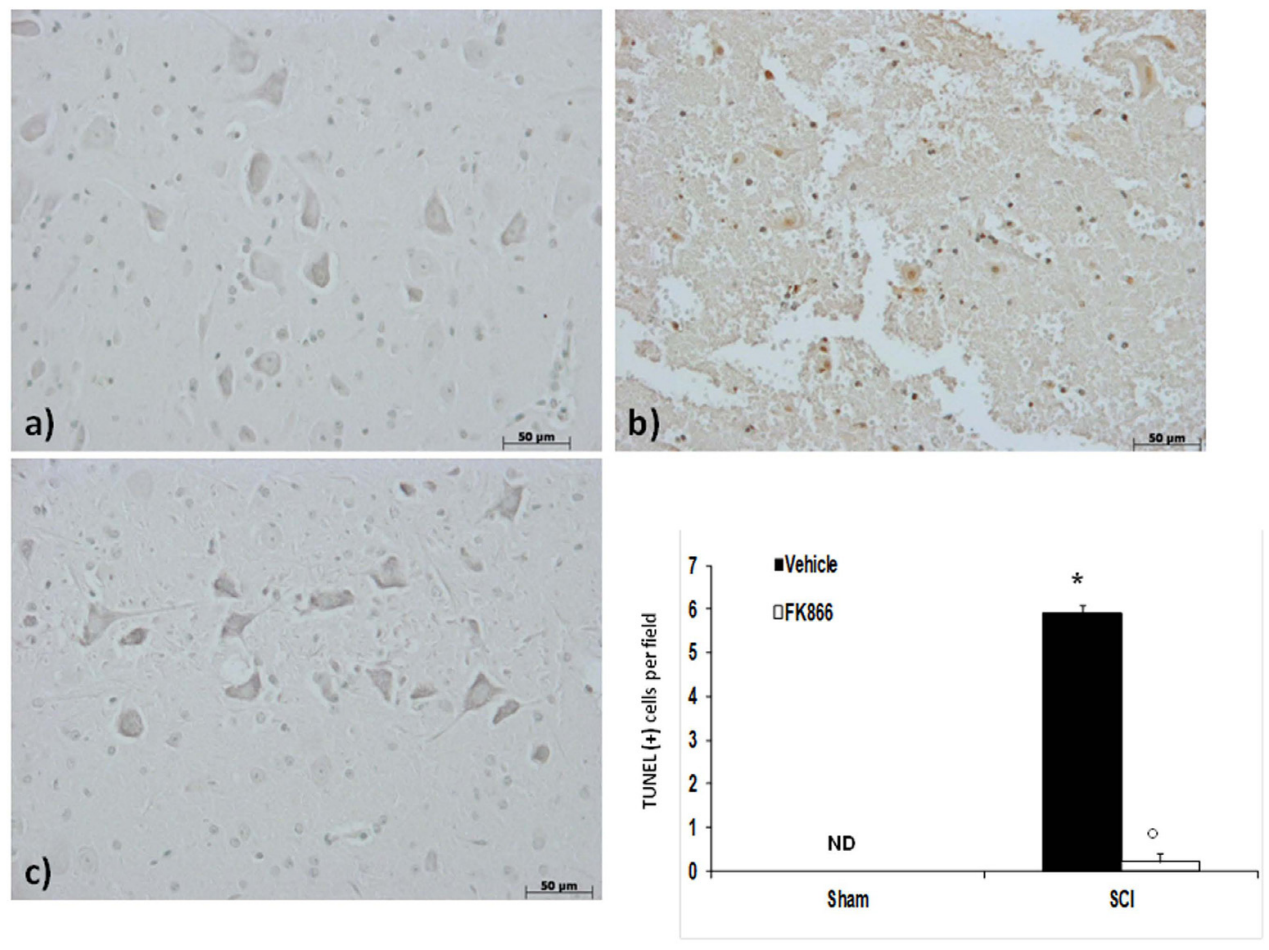
**Effects of FK866 on TUNEL-like staining in the perilesional spinal cord tissue**. At 24 h after the trauma, SCI mice demonstrated a marked appearance of dark brown apoptotic cells and intercellular apoptotic fragments (**b**). In contrast, tissues obtained from mice treated with FK866 (**c**) no revealed apoptotic cells or fragments. The number of TUNEL positive cells/high-power field was counted in 5 to 10 fields for each coded slide. The count of TUNEL cells per field was made by an independent observer. This figure is representative of at least three experiments performed on different experimental days. wm, white matter; gm, gray matter. Values shown are mean *± *S.E. mean of 10 mice for each group. *p < 0.01 vs sham, °*p *< 0.01 vs SCI + vehicle.

No apoptotic cells were detected in the spinal cord from sham-operated mice (Figure 8a). At 24 h after the trauma, tissues from SCI mice demonstrated a marked appearance of dark brown apoptotic cells and intercellular apoptotic fragments (Figure 8b). In contrast, tissues obtained from mice treated with FK866 demonstrated no apoptotic cells or fragments (Figure 8c and densitometric analysis).

### Effect of FK866 on neurotrophic factors

**Figure 9:**
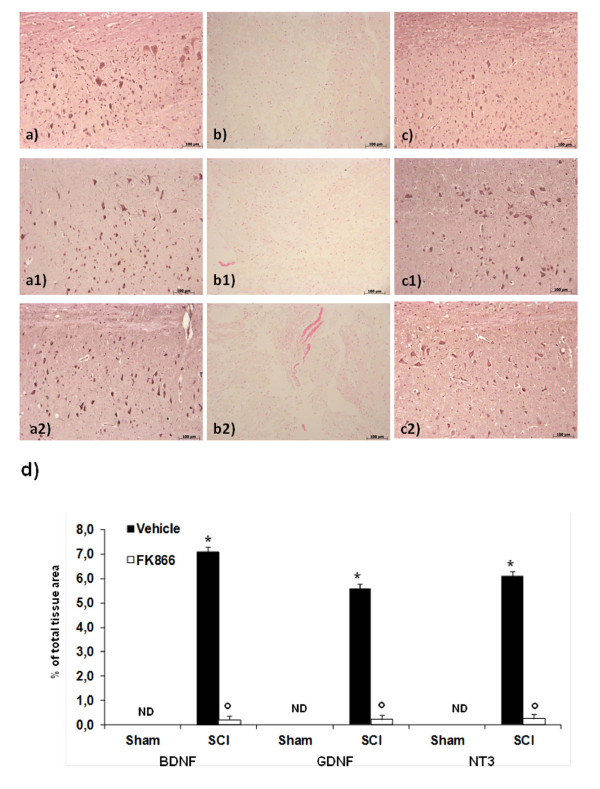
**Effects of FK866 on neurotrophins expression**. Spinal cord sections were processed at 24 h after SCI to determine the levels for BDNF, GDNF and NT3 by immunohistochemical analysis. A basal level of BDNF (**a**), GDNF (a1) and NT3 (**a2**) were detected in the spinal cord from Sham-operated animals, whereas BDNF, GDNF and NT3 levels were substantially reduced in SCI mice **(b, b1, b2 respectively)**. FK866 (**c, c1, c2 **respectively) treatment prevented the SCI-induced BDNF, GDNF and NT3 reduction. In panel d densitometric analysis is reported. *p < 0.01 vs sham, °*p *< 0.01 vs SCI + vehicle.

To test whether FK866 modulates the inflammatory process through regulation of neutrophic factors, we have examined BDNF, GDNF and NT-3 levels in the perilesioned zone both by immunohistochemistry. In the spinal cord tissues collected at 24 h after the trauma, neurotrophic factors expression levels were significantly reduced (Figure 9b, b1 and b2) in comparison to sham animals (Figure 9a, a1 and a2). The treatment with FK866 (Figure 9c, c1, c2, and densitometric analysis) significantly restored the levels of the three neurotrophic factors up to that of uninjured mice.

### FK866 treatment reduces astrocyte and microglial activation after SCI

**Figure 10:**
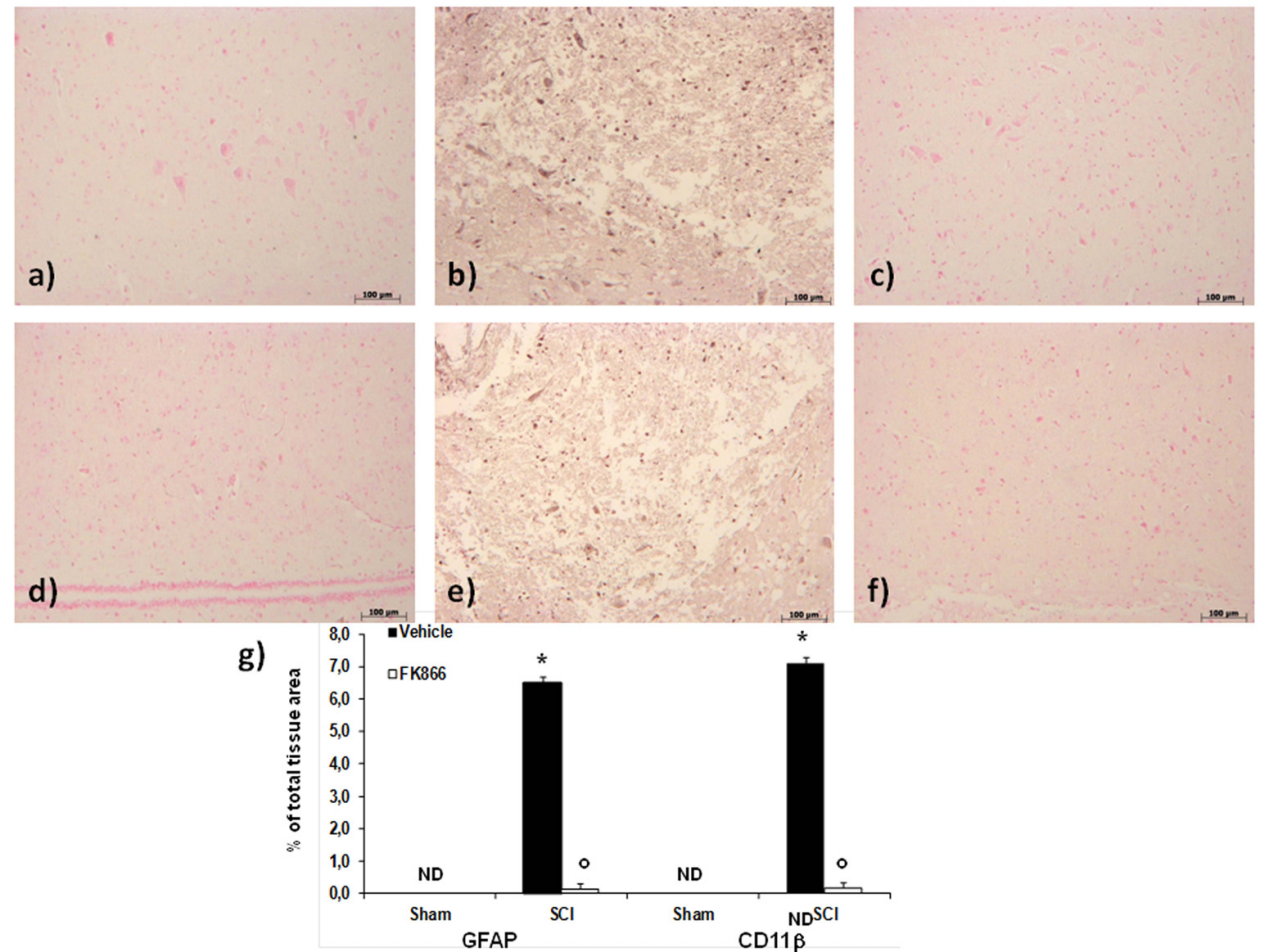
**FK866 treatment reduces astrocyte and microglial activation after SCI**. Spinal cord sections revealed increased astrogliosis (GFAP + cells) in the perilesional area after SCI (**b**). On the contrary, a significant number of non-GFAP positive cells were found in the spinal cord from FK866 (**c**) treated mice. Moreover, microglial cells were activated following SCI as shown by increased CD11b-positive staining (**e**). Expression of CD11b was attenuated by FK866 (**f**) treatment. In panel **g **densitometric analysis is reported. *p < 0.01 vs sham, °*p *< 0.01 vs SCI + vehicle.

Analysis was performed to elucidate the nature of cells targeting by FK866. Spinal cord sections revealed increased astrogliosis (GFAP + cells) in the perilesional area after SCI (Figure 10b). On the contrary, a significant number of non-GFAP positive cells were found in the spinal cord from FK866-treated mice (Figure 10c). Moreover, microglial cells were activated following SCI as shown by increased CD11b-positive staining (Figure 10e). Expression of CD11b were attenuated by FK866 (Figure 10f, and densitometric analysis).

## Conclusions

SCI can result in severe disability, and it is at present a disease with a high therapeutic unmet need. The pathophysiology of SCI can be divided in two separate distinct phases: a primary injury elicited by the compression that determines a patient's prognosis and a secondary phase which can exacerbate damage and limit restorative processes, and therefore, contribute to overall morbidity and mortality. Unambiguously, inflammatory response following SCI contributes substantially to these secondary devastating effects. SCI-induced inflammation can lead to further reduction in functional recovery because of the development of scar tissue, as well as necrosis or apoptosis of neurons and myelin forming oligodendrocytes [36].

In the present manuscript, we have investigated the effect of the inhibition of an enzyme involved in NAD metabolism, NAMPT, in SCI recovery. NAMPT exists in two major forms, an intracellular one which has been implicated directly in NAD homeostasis, and an extracellular one, which is also named visfatin or PBEF [3]. Whether visfatin exerts its functions via modulating NAD homeostasis is at present unclear, and a number of other mechanisms have been proposed [37]; [38]. Furthermore, it cannot be ruled out that extracellular NMN, the product of NAMPT, may have biological functions. What is beyond doubt is that the extracellular form of visfatin displays plasma increases in a number of human inflammatory diseases.

We now show that NAMPT protein levels are increased upon SCI in the peri-lesional area. This could be due to the invasion of activated immune cells with upregulated NAMPT expression [15] into the injured spinal tissue. More importantly, we show for the first time that NAMPT inhibition corrects elevated NAMPT levels and ablates various inflammatory aspects of SCI-associated secondary damage by targeting neutrophils, macrophages, microglia and astrocytes, thereby leading to a significant recovery of the motor performance of injured animals.

Neutrophils are the first inflammatory cells to arrive at the site of injury and are implicated in the modulation of the secondary injury by release of reactive mediators that damage endothelial cells and increase vascular permeability thereby exaggerating immune cell invasion to the spinal cord leading to further damage [39]. Neutrophils infiltration, as measured by MPO activity was significantly increased 24 h post trauma in spinal cord. In FK866-treated animals, SCI-evoked elevated MPO level was profoundly decreased and was accompanied by an overall attenuation of inflammatory mediators and motor function in mice.

Microglia and macrophages dominate the innate immune response elicited by SCI and sustain the secondary damage in animal models of SCI [20] as macrophage activation and their cytokine release, notably TNF-α and IL-1β, contribute to axonal demyelination [40] and cell death [41]. Twenty-four h after traumatic SCI, spinal content of the proinflammatory TNF-α and IL-1β as well as CD11b positive cells displayed a substantial rise, while NAMPT inhibition by administration of FK866 prevented the elevation of inflammatory markers. These findings paralleled the reversal by FK866 of SCI-induced myelin degradation in spinal cord tissues as verified by less myelin phagocytosis by macrophages in Oil Red staining and less degenerating myelin in Luxol fast blue staining. Moreover, in spinal cord sections from injured-mice a dramatic astrogliosis denoted by increased immunoreactivity of GFAP was observed. As reactive astrocytes gives rise to an astroglial scar that acts as a physical and/or chemical barrier to axonal regeneration [42], its inhibition achieved by administration of FK866 is beneficial as was associated with more intact white matter in histologic evaluation.

Apoptosis is another component of secondary injury that can be triggered by a variety of insults including cytokines, inflammatory injury and free radical damage. Apoptosis in oligodendrocytes contributes to post-SCI demyelination while apoptosis in neurons contributes to cell loss [43,44]. In our study, apoptosis was evident in both white and gray matter of injured spinal cord as evidenced by TUNEL positive cells and augmented tissue Bax/Bcl-2 ratio. Interestingly in FK866-treated mice, no apoptosis was detected, pro-apoptotic Bax significantly decreased and anti-apoptotic Bcl-2 restored. These observations were accompanied by the complete replenishment of the neurotrophic factors BDNF, GDNF and NT-3 as well as amelioration of neuron morphology in perilesional spinal cord sections from mice receiving FK866.

The beneficial properties of FK866 observed herein would be in accord with recent reports indicating that NAMPT inhibitors can ameliorate animal model symptomatology of inflammatory diseases such as arthritis, endotoxic shock and autoimmune encephalitis [15-17]. While the mechanism by which this class of drugs act as anti-tumoral agents is thought to involve energy deprivation [36], how FK866 should reduce inflammation is less straightforward. It has been recently shown that a reduction of PARP and/or SIRT activity might in part explain this phenomenon [17]. Indeed, this would also be conceivable in SCI. PARP is a ubiquitous, chromatin-bound enzyme abundantly present in the nuclei of numerous cell types [24](Szabó and Dawson, 1998). Continuous or excessive activation of PARP produces extended chains of ADP-ribose on nuclear proteins and results in a substantial depletion of intracellular NAD^+ ^and subsequently, adenosine triphosphate (ATP), leading to cellular dysfunction and ultimately, cell death [45]. Consistent with our observation of a drastic rise in PAR level, an indicator of PARP activation, 24 h following SCI it has been demonstrated that SCI induces PARP activation [25] and that pharmacologic or genetic ablation of PARP activity can reduce the degree of tissue injury associated with spinal cord trauma [46]. Of note, treatment with FK866 blunted the SCI-evoked PARP activation indicating PARP as a potential signaling pathway targeted by NAMPT inhibitors to elicit their neuroprotection. SIRTs constitute another group of NAD-dependent enzymes with divergent roles in neuronal survival. SIRT1 elicits anti-apoptotic effects while SIRT2, 3 and 6 have been shown to promote cell death [47]. Specifically, SIRT2 is reportedly increased upon damage to the spinal cord in rat following a proteomic approach, while its pharmacological or genetic inhibition protects against neurotoxicity in models of Parkinson's disease [48]. Therefore, it is plausible that NAD depletion following administration of NAMPT inhibitors decreases SIRT2 deacetylase activity and confers protection against deleterious stimuli in SCI. Of importance, recent evidence also suggests that cell survival promoting properties of SIRT1 might be mediated by a non-catalytic mechanism and, hence, can be preserved in presence of NAMPT inhibitors.

While the processes downstream of NAD depletion have not been evaluated in the present manuscript, we have observed that (i) inflammatory cytokines (eg. TNF-α and IL-1β) are increased upon SCI and are significantly reduced by NAMPT inhibition; (ii) NF-κB expression was also significantly increased upon SCI and normalized by NAMPT inhibition and (iii) that neutrophil infiltration and reactive gliosis, two hallmarks of the inflammatory process which takes place in the injured spinal cord, were significantly reduced by FK866 treatment. These data, taken together, would suggest that the inflammatory component of the injury is the primary target of these inhibitors. FK866 could (also) alleviate SCI by inhibiting TNF-α secretion by macrophages and microglia, thereby reducing inflammation and thus preventing the damage.

This study suggests that FK866, a specific inhibitor of NAMPT, administered after SCI, is capable of reducing the secondary injury and partly reduce permanent damage. The specificity of the effect is supported by the fact that another inhibitor of NAMPT, termed GPP78, elicits the same effects. Both drugs have been administered after the trauma was elicited, in a setting that mimics the clinical situation, and therefore our results may have clinical implications.

## Competing interests

The authors declare that they have no competing interests.

## Authors' contributions

EE: conceived of the study, helped to draft the manuscript. DI: carried out Western blot analysis. GF and RR: carried out Western blot and immunoassays. EM: performed immunohistochemical localization. CT: performed acquisition and analysis of data. GCT: synthetized chemical compounds. SC and AAG: participated in the conception and design of experiments, analysis and interpretation of data. All authors read and approved the final manuscript.
